# Exploring neural correlates of automated speech-based cognitive markers through resting-state functional connectivity in aging and at-risk Alzheimer’s disease

**DOI:** 10.1186/s13195-026-01993-x

**Published:** 2026-02-24

**Authors:** Qingyue Li, Zampeta-Sofia Alexopoulou, Martin Dyrba, Elisa Mallick, Johannes Tröger, Eike Spruth, Slawek Altenstein, Claudia Bartels, Wenzel Glanz, Enise I. Incesoy, Michaela Butryn, Ingo Kilimann, Sebastian Sodenkamp, Franziska Maier, Ayda Rostamzadeh, Antje Osterrath, Josef Priller, Anja Schneider, Jens Wiltfang, Christoph Laske, Björn Falkenburger, Michael Wagner, Emrah Duezel, Annika Spottke, Gabor C. Petzold, Frank Jessen, Alexandra König, Stefanie Köhler, Stefan Teipel, DELCODE, DESCRIBE study groups

**Affiliations:** 1 Department of Psychosomatic Medicine, Rostock University Medical Center, Gehlsheimer Str. 20, Rostock, 18147 Germany; 2 Cognition-Behavior-Technology Lab (CoBtek), Université Côte d’Azur, Nice, France; 3 German Center for Neurodegenerative Diseases (DZNE), Rostock, Germany; 4 ki:Elements GmbH, Saarbrücken, Germany; 5 German Center for Neurodegenerative Diseases (DZNE), Berlin, Germany; 6 Department of Psychiatry and Psychotherapy, Charité, Berlin, Germany; 7 Department of Psychiatry and Psychotherapy, University Medical Center Göttingen, University of Göttingen, Göttingen, Germany; 8 German Center for Neurodegenerative Diseases (DZNE), Magdeburg, Germany; 9 Institute of Cognitive Neurology and Dementia Research (IKND), Otto-Von-Guericke University, Magdeburg, Germany; 10 German Center for Neurodegenerative Diseases (DZNE), Tübingen, Germany; 11 Department of Psychiatry and Psychotherapy, University of Tübingen, Tübingen, Germany; 12 Department of Psychiatry, Medical Faculty, University of Cologne, Cologne, Germany; 13 German Center for Neurodegenerative Diseases (DZNE), Dresden, Germany; 14 Department of Neurology, Carl Gustav Carus, Tec, University Hospital, hnische Universität Dresden, Dresden, Germany; 15 University of Edinburgh and UK DRI, Edinburgh, UK; 16 Department of Psychiatry and Psychotherapy, School of Medicine and Health, Technical University of Munich, and German Center for Mental Health (DZPG), Munich, Germany; 17 German Center for Neurodegenerative Diseases (DZNE), Bonn, Germany; 18 Clinic of Old Age Psychiatry and Cognitive Disorders, University Hospital Bonn and University of Bonn, Bonn, Germany; 19 German Center for Neurodegenerative Diseases (DZNE), Göttingen, Germany; 20 Department of Medical Sciences, Neurosciences and Signaling Group, Institute of Biomedicine (iBiMED), University of Aveiro, Aveiro, Portugal; 21 Clinic for Parkinson’s, Sleep and Movement Disorders, Centre for Neurology, University Hospital Bonn, Bonn, Germany; 22 Department of Vascular Neurology, University Hospital Bonn, Bonn, Germany; 23 Excellence Cluster On Cellular Stress Responses in Aging-Associated Diseases (CECAD), University of Cologne, Cologne, Germany; 24 Centre Hospitalier Et Universitaire, Clinique Gériatrique du Cerveau Et du Mouvement, Centre Mémoire de Ressources Et de Recherche, Université Côte d’Azur, Nice, France

**Keywords:** Functional MRI, Multicenter, Speech, Digital assessment, Alzheimer’s disease spectrum

## Abstract

**Background:**

Digital speech-based assessments provide scalable tools for detecting subtle cognitive decline. Here, we investigated whether digitally derived speech-based composite score of cognition and individual speech features were associated with alterations in functional connectivity (FC) within task-related brain networks in the Alzheimer’s disease spectrum, which are known to reflect cognitive performance and disease-related changes.

**Methods:**

Data were analyzed from 129 participants of the German PROSPECT-AD study, ranging from cognitively healthy individuals to those with mild cognitive impairment. Speech-based cognitive scores and speech features were derived from automated phone-administered semantic verbal fluency (SVF) and verbal learning tasks (VLT). Resting-state fMRI assessed FC, with intrinsic connectivity networks identified via independent component analysis and dual regression. Associations were examined using permutation-based voxel-wise regression, controlling for demographic and clinical covariates. Seed-to-voxel analyses were conducted to support network identification and complement findings.

**Results:**

Greater language network connectivity in the left middle temporal gyrus was associated with increased SVF temporal cluster switching (FWE < .05, cluster size = 12 voxels, mean T = 3.86). Exploratory analyses (uncorrected *p <* .01) demonstrated no significant associations between cognitive composite scores and FC. However, individual SVF and VLT speech features exhibited network-specific associations across executive, language, and default mode networks, indicating exploratory yet spatially distinct connectivity patterns.

**Conclusion:**

Digital speech-based assessments may have limited current utility for detecting FC alterations in at-risk individuals. Further validation using complementary methodological approaches, shorter intervals between fMRI and speech assessments, and testing in independent cohorts, are essential to establish their reliability and clinical relevance for monitoring brain network changes.

**Supplementary Information:**

The online version contains supplementary material available at 10.1186/s13195-026-01993-x.

## Background

As prevention-focused and combinatorial therapeutic strategies to address the pre-symptomatic stages of Alzheimer's disease (AD) are being developed, those with mild cognitive impairment (MCI), as well as cognitively normal individuals who might be at risk of developing AD, are increasingly taking part in clinical trials [1]. This trend underscores the need for easy-to-use and widely available tools for tracking cognition, where they might be used to identify risk in clinical care or targeted into clinical trials [2]. Digital cognitive tests for this purpose are a promising approach that is becoming more and more accepted, particularly in preclinical and prodromal AD research studies [3, 4]. These remote automated methods can both improve efficiency and reduce the burden on-site, whilst enabling the recruitment of participants from a broader and more diverse range of groups [4, 5].

Assessment based on verbal response is especially valuable because it can capture subtle linguistic and acoustic changes that often occur in early AD [6, 7]. Semantic Verbal Fluency (SVF) for testing executive function and semantic retrieval [8], and the Verbal Learning Task (VLT) for assessing episodic memory [9], are frequently used tasks for speech feature extraction. Speech features from these two tasks can identify subtle cognitive changes that usually remain unnoticed by global cognitive measures [10, 11]. Moreover, distinguishing subjective cognitive decline (SCD) from MCI/AD has become more accurate when speech features and task scores are combined [12, 13]. These features have also been related to traditional neuropsychological scores and established AD biomarkers, including amyloid beta (Aβ) and phosphorylated tau levels [14, 15]. Notably, it has recently been shown that cognitive composite scores that are exclusively computed from these speech features were correlated with standardized neuropsychological results, such as the validated speech-based cognitive (SB-C) scores [16]. It has also been reported that the SB-C scores were associated with longitudinal changes in cognition and brain atrophy [17]. But we are not yet sure whether these speech-derived indicators can signify other brain pathologies beyond volumetric alteration. Closing this gap could help clarify the extent to which speech-based tools can serve as a window into broader brain dysfunctions related to AD progression.

Of the various modalities, functional connectivity (FC) is a particularly good option for examination. Changed FC has also been considered a sensitive neuroimaging marker for early AD diagnosis, with the potential to be identified even before the onset of clinical cognitive deficits [18, 19]. Resting-state functional MRI (rs-fMRI), which does not require subjects to perform any tasks while scanning, is widely used to measure FC. Accordingly, this method is particularly appropriate for studies involving populations with cognitive impairments [20, 21]. By using rs-fMRI, researchers can investigate global FC at the network level to understand how different regions of the brain interact with one another. Research on AD has investigated several well-defined networks, including the default mode network (DMN), the executive control network (ECN), and the language network (LAN) [19, 22–26]. From all results, changes in the connectivity of the DMN have emerged as one of the most consistent findings in studies involving both AD and MCI groups [23, 27, 28]. This disruption in the DMN has also been associated with memory issues [23, 29, 30] and an increased risk of disease progression [31]. Research also showed that ECN connectivity alterations were correlated with executive dysfunction, manifested by abnormalities in the lateral prefrontal cortex and its connections with parietal and subcortical regions. Significant differences have been identified between individuals with MCI/AD and healthy people [23, 24, 26]. Changes in FC in the LAN have also been reported, showing links to semantic processing and connected speech deficits, with evidence showing that alterations in network-specific connectivity may support language performance in early phases of disease [25, 32].

Building on this rationale, the present study investigated how SB-C scores and key speech features associated with FC within related networks were affected in at-risk AD populations, including the DMN, ECN, and LAN. Our hypotheses were grounded in prior evidence linking specific cognitive abilities, as measured by speech-based tasks, to distinct neural networks. For this purpose, we examined (1) the associations between SB-C scores and connectivity patterns across these three networks; (2) the associations between SVF features and connectivity in the ECN and LAN; and (3) the associations between VLT delayed recall features and DMN connectivity. This network-specific approach aimed to deepen our understanding of how digital speech markers correspond to potential underlying changes of the neural networks, providing evidence for their possible use as objective proxies for brain alterations and informing the development of sensitive, scalable tools for detecting cognitive decline risk.

## Methods

### Recruitment and sample

We used data from the German arm of the PROSPECT-AD study, a multicenter prospective initiative aimed at validating speech-based markers for the early detection and monitoring of AD [33]. The study design and protocol have been described in detail [33]. PROSPECT-AD integrates automated speech-based neurocognitive assessments into ongoing longitudinal cohorts, including DESCRIBE and DELCODE in Germany [34].

Participants aged 50 years or older were recruited from the DESCRIBE and DELCODE cohorts, representing a continuum of risk for developing AD dementia. Exclusion criteria were unstable medical conditions, major psychiatric disorders, or substance abuse. Of the 234 initially enrolled participants, 10 were excluded due to incomplete speech assessments. An additional group of participants was excluded due to the absence of qualified MRI scans, resulting in a final sample of 132 individuals. This sample comprised healthy controls (HC) and participants with SCD or MCI. The informed consent of all participants was obtained, and local ethics committees granted approval for the procedures.

Site clinicians determined clinical diagnoses based on structured clinical interviews, neuropsychological assessments, and established diagnostic criteria as follows. The HC group included individuals without cognitive complaints or objective impairment, as well as cognitively healthy first-degree relatives of AD patients. SCD was defined as persistent self-reported cognitive decline without objective impairment [35], while MCI was diagnosed according to the NIA criteria [36, 37].

### Speech features collection, processing, and selection

Speech assessments were conducted remotely using the automated phonebot ‘Mili’ (ki:elements GmbH, Germany, https://ki-elements.de/mili-platform/). Participants were asked to complete six phone assessments over 15 months. The current analysis focused on data collected during the first call. Each speech assessment lasted approximately 15 min and included the following tasks: VLT (15-word, across 4 encoding trials), SVF (animal category, 1 min), a one-minute storytelling task describing a positive life event, and the delayed recall of the VLT at the end.

**Fig. 1:**
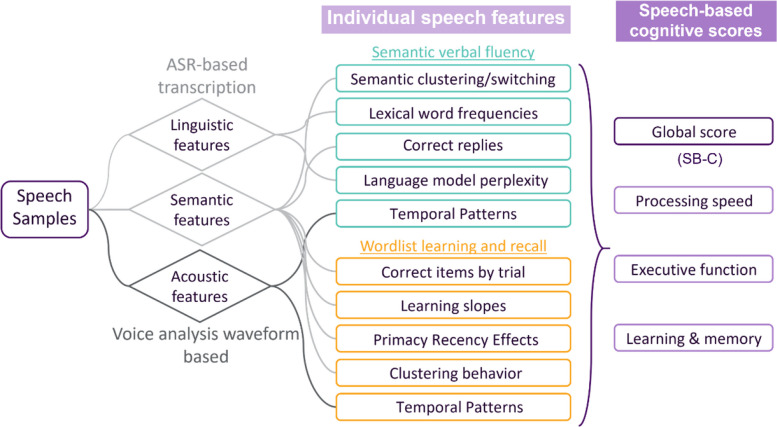
Schematic representation of the processing steps involved in collecting and calculating the SB-C scores

Speech recordings from the VLT and SVF tasks were processed using ki:elements’ proprietary SIGMA speech analysis pipeline [38, 39]. This included German-optimized automatic speech recognition, transcription, voice analysis, and feature extraction. Based on speech features, the SB-C global score and three domain-specific SB-C subscores (executive function, learning and memory, and processing speed) were computed (Fig. 1).

Besides the SB-C global and subscores, we selected a hypothesis-driven subset of speech features based on their theoretical relevance to detect subtle cognitive changes sensitively. For the SVF task, we used correct replies (i.e., correct count) as a general measure, alongside lexical-semantic clustering/switching features including semantic cluster size and switch counts, which have established associations with semantic memory and executive function [11]. Word frequency was included because it relates to early word-finding difficulties and changes in word production in AD [40]. Temporal features, including temporal cluster size and switch counts, which complemented semantic features by measuring patterns of exploitation and exploration [42], were also included. For the VLT, we focused exclusively on delayed recall features, which comprised correct recall counts, word position-based measures (primacy, midlist, recency), and serial clustering size. This focus is supported by evidence that poor delayed recall performance is one of the early signs of episodic memory impairment and progression to AD [55]. Among the selected features, serial clustering size was chosen to capture sequential recall patterns linked to episodic memory organization [43]. Additionally, poorer recall of early list items during delayed recall, known as a diminished primacy effect, has been identified as a sensitive predictor of subsequent cognitive decline in AD [10]. All selected features, except primacy items, have also been identified among top speech recognition markers differentiating SCD from MCI/dementia [12, 13]. The full list of selected features and their descriptions is provided in the supplementary material (Table S1).

### Imaging acquisition and processing

Imaging data were acquired using identical acquisition parameters and harmonized protocols from nine Siemens 3.0 Tesla MRI scanners (4 Verio, 1 Skyra, 3 TimTrio, and 1 Prisma system) at all study sites. To keep the data quality uniform, a semi-automated quality control process was implemented during the acquisition phase, with feedback provided to sites in case of protocol deviations. High-resolution T1-weighted structural MRI was obtained using a sagittal magnetization-prepared rapid gradient echo (MPRAGE) sequence with the following parameters: matrix size = 256 × 256, number of slices = 192, field of view = 256 × 256 mm, isotropic voxel size = 1 mm, repetition time = 2500 ms, echo time = 4.37 ms, flip angle = 7°, and parallel imaging acceleration factor = 2. Rs-fMRI data were collected using a T2*-weighted echo-planar imaging (EPI) sequence with the following parameters: matrix size = 64 × 64, 47 axial slices (thickness 3.5 mm, no gap) with interleaved acquisition, field of view = 224 × 224 × 165 mm, isotropic voxel size = 3.5 mm, repetition time = 2580 ms, echo time = 30 ms, flip angle = 80°, and parallel imaging acceleration factor = 2. A total of 180 EPI volumes were acquired over an 8-min scan.

There was no systematic approach to synchronize imaging and speech assessment because both were part of ongoing longitudinal studies. Therefore, for the purpose of minimizing the temporal mismatch, the closest MRI scan to a speech assessment was chosen for each subject.

Data processing was carried out using Data Processing Assistant for Resting-State fMRI Advanced (DPARSFA 5.3) [44] implemented in MATLAB R2020a (MathWorks, Natick, MA). The first 10 time points were discarded to allow for magnetic field stabilization and participant adaptation. Rs-fMRI data underwent slice-timing correction and realignment to the mean functional image to correct for head motion. During realignment, all functional images were resliced to an isotropic voxel size of 3× 3 × 3 mm. The T1-weighted anatomical images were segmented into gray matter, white matter, and cerebrospinal fluid using the segmentation function within DPARSFA, which utilizes SPM12 (Statistical Parametric Mapping, Wellcome Trust Centre for Neuroimaging, London, UK, http://www.fil.ion.ucl.ac.uk/spm/), *New Segment* toolbox. Spatial normalization to Montreal Neurological Institute (MNI) space was performed using deformation fields generated by DARTEL (Diffeomorphic Anatomical Registration Through Exponentiated Lie Algebra) [45]. Residual head motion was evaluated by calculating the mean frame-wise displacement [46], with 0.5 mm being the cutoff for exclusion. After normalization, functional data were band-pass filtered (0.01–0.1 Hz) and smoothed using Gaussian kernels of varying full-width at half-maximum (FWHM) (see Sect. " Independent component analysis and dual regression" for details of smoothed kernels).

### Independent component analysis and dual regression

We applied a single-group independent component analysis (ICA) by pooling all qualified resting-state scans across diagnostic groups to derive intrinsic connectivity networks that reflect a shared functional architecture across participants. This approach provided a common spatial reference for subsequent association analyses, ensuring that relationships between speech features and functional connectivity can be mapped consistently across individuals spanning the AD-risk spectrum. The resting-state FC (rsFC) maps were calculated using FSL (Version 5.0.9, FMRIB, Oxford, UK, http://www.fmrib.ox.ac.uk/fsl/) *melodic* toolbox, resulting in group-level ICA maps. As no standardized criteria exist for optimal smoothing kernels or ICA dimensionalities in the literature [47, 48], we systematically tested combinations of preprocessing parameters, including spatial smoothing kernels of 6, 8, 10 mm, and ICA with 10, 20, 25, 30, and 40 components. The optimal combination was selected based on three criteria: (1) the spatial stability across runs, (2) the least inter-component correlations, and (3) the moderate-to-high spatial overlap with established functional templates. Of the different combinations we tested, the solution with an 8 mm smoothing kernel and 40-component ICA best met our criteria. Importantly, this configuration enabled the reliable identification of the LAN, which was not consistently detectable at lower ICA dimensionalities.

The generated independent components (ICs) were evaluated independently by two trained researchers and confirmed by experts to identify the three resting-state networks (RSNs) of interest, including DMN, ECN, and LAN. We used the *FSLcc* tool to measure correlations between ICs and established network templates [49, 50]. In addition, the ICs were visually inspected to select those covering key regions involved in each network, including the left inferior frontal gyrus and left posterior superior temporal gyrus for LAN, the posterior cingulate cortex (PCC) for DMN, and the medial prefrontal cortex for ECN. Identified ICs were also compared across multiple ICA configurations to assess the stability and consistency of the derived networks. For ICs showing spatial variability, we conducted sensitivity analyses to evaluate whether the ICA pooling strategy affected our primary results (Supplement 1). Subject-level rsFC z-maps were estimated using *dual regression* implemented in FSL, following ICA. This included (1) regressing the group-level spatial maps into each subject’s 4D dataset to extract individual time courses, and (2) regressing these time courses back into the same dataset to obtain subject-level spatial maps for each RSN [51, 52]. We then performed a subsequent analysis to check whether these spatial maps were statistically different between diagnostic groups (Supplement 1, Table S5). Overall, the spatial topography of the DMN, ECN, and LAN did not differ significantly between diagnostic groups, confirming that the networks are representative of all participants.

For subsequent regression analyses, explicit masks for each RSN of interest were created by thresholding and binarizing the group-level ICs. DMN and ECN were thresholded at the top 10% of voxel intensities [23], while the LAN was thresholded using a T-value cutoff (peak T > 5.0) to achieve a better spatial specificity.

### Statistical analysis

Statistical analyses were conducted using R version 4.4.2 [53], with functions from the *stats* package, and the non-parametric permutation-based tool *randomize* implemented in FSL. Group differences in demographic variables and speech features were assessed using one-way ANOVA with Tukey’s honest significant difference (HSD) post-hoc tests. For variables that violated normality assumptions, Kruskal–Wallis tests were applied, and chi-squared tests were used for categorical variables. Statistical significance was set at *p <* 0.05.

Voxel-wise multiple regressions were performed using general linear models with 5,000 permutations. Speech features and SB-C scores were regressed on the rsFC z-maps hypothesized to be relevant. SVF word frequency was modeled to detect negative associations with rsFC, while all other speech features were modeled for positive associations. Covariates included age, sex, education, diagnosis, scan site, and the time interval between speech assessment and MRI acquisition to account for potential temporal mismatch.

To examine potential group differences in the associations between speech-derived measures and functional connectivity, we additionally fitted models including speech feature × diagnosis interaction terms within the primary ICA-based framework. Sensitivity analyses employing a complementary seed-to-voxel approach were conducted to validate the ICA-derived network definitions and replicate the across-group association results (see Supplement 2 for full details).

Statistical significance was determined using Threshold-Free Cluster Enhancement (TFCE) with family-wise error (FWE) correction at *p <* 0.05 [54]. When no clusters survived correction, a more liberal descriptive threshold of uncorrected *p <* 0.01 with a minimum cluster size of 20 voxels was applied. To ensure that identified effects reflected network-level connectivity patterns, significant voxel-wise results were mapped onto our predefined group-level RSN masks derived from ICA and visualized using *MRIcroGL* (https://www.nitrc.org/projects/mricrogl/.). A chord diagram was generated in R using the *circlize* package, illustrating the number of significant voxels and the strength of associations (T-values) between speech features and RSN regions.

## Results

### Demographic and speech characteristics

After excluding 3 participants for excessive head motion, the final sample included 129 individuals (32 HC, 72 SCD, 25 MCI; mean age = 73 years; 55% female; mean educatio*N =* 15 years; mean time interval between speech assessment and MRI acquisitio*N =* 1.4 years). Diagnostic groups did not differ significantly in age, education, or time interval between speech assessment and MRI acquisition, but differed in sex distribution (χ^2^ = 7.12, *p =* 0.03). Significant group differences were found across SB-C scores and subdomain scores, with post-hoc tests indicating lower performance in the MCI group. Several speech features, including SVF correct count, semantic cluster size, and VLT delayed recall, also differed significantly across groups (Table 1).

**Table 1:** Sample description of the dataset

		**Overall**	**HC**	**SCD**	**MCI**	**Group difference**
	**Mean (SD)**	**(*N =* 129)**	**(*N =* 32)**	**(*N =* 72)**	**(*N =* 25)**	***Post-hoc***
**Demographic**	**Age at scan**	72.8 (6.57)	73.4 (5.83)	72.3 (6.61)	73.7 (7.42)	F(2, 126) = 0.51, *p =* 0.60^a^
	**Sex (f, %)**	71 (55%)	24 (75%)	36 (50%)	11 (44%)	**X**^**2**^**(2) = 7.12, *p =* 0.03**^**b**^
	**Education (years)**	14.9 (2.68)	14.8 (2.62)	15.1 (2.67)	14.3 (2.82)	H(2) = 2.18, *p =* 0.337^c^
	**Number of scan sites**	8	3	7	6	
	**Time interval (years)**	1.38 (2.22)	2.27 (2.98)	1.15 (1.89)	0.89 (1.69)	H(2) = 1.53, *p =* 0.47^c^
	**MMSE score**^**d**^	29.0 (1.41)	29.2 (1.26)	29.4 (0.98)	27.9 (2.01)	**H(2) = 15.05, *p <* 0.001**^**c**^
						MCI-HC*
						MCI-SCD***
**SB-C global**	**Global cognition score**	0.67 (0.25)	0.74 (0.27)	0.70 (0.19)	0.49 (0.28)	**F(2, 126) = 9.86, *p <* 0.001**^**a**^
						MCI-HC***
						MCI-SCD***
**SB-C subdomain**	**Executive score**	0.50 (0.22)	0.53 (0.22)	0.53 (0.20)	0.38 (0.23)	**F(2, 126) = 5.38, *p <* 0.01**^**a**^
						MCI-HC*
						MCI-SCD**
	**Memory score**	0.57 (0.20)	0.61 (0.21)	0.59 (0.16)	0.44 (0.23)	**H(2) = 10.61, *p <* 0.01**^**c**^
						MCI-HC**
						MCI-SCD*
	**Processing speed score**	0.74 (0.31)	0.83 (0.34)	0.77 (0.24)	0.57 (0.36)	**F(2, 126) = 6.00, *p <* 0.01**^**a**^
						MCI-HC**
						MCI-SCD*
**SVF**	**Correct count**	20.3 (6.80)	20.4 (6.51)	21.9 (6.10)	15.7 (7.21)	**F(2, 126) = 8.63, *p <* 0.001**^**a**^
						MCI-HC*
						MCI-SCD***
	**Word frequency**	3.63 (0.25)	3.62 (0.28)	3.59 (0.20)	3.76 (0.28)	**F(2, 126) = 4.66, *p =* 0.01**^**a**^
						MCI-SCD**
	**Semantic cluster size**	3.54 (1.29)	3.41 (1.14)	3.84 (1.33)	2.86 (1.07)	**H(2) = 13.68, *p <* 0.001**^**c**^
						MCI-SCD***
	**Semantic cluster switches**	5.37 (2.02)	5.63 (1.84)	5.35 (2.14)	5.12 (1.90)	H(2) = 0.84, *p =* 0.66^c^
	**Temporal cluster size**	4.19 (1.98)	4.63 (2.44)	4.10 (1.64)	3.89 (2.20)	H(2) = 3.21, *p =* 0.20^c^
	**Temporal cluster switches**	4.60 (2.07)	4.28 (1.85)	4.99 (2.05)	3.92 (2.20)	**F(2, 126) = 3.09, *p =* 0.049**^**a**^
**VLT delayed recall**	**Correct count**	10.3 (3.44)	10.9 (2.88)	11.2 (2.64)	7.08 (4.27)	**H(2) = 19.53, *p <* 0.001**^**c**^
						MCI-HC**
						MCI-SCD***
	**Midlist items**	4.91 (1.81)	5.22 (1.68)	5.29 (1.45)	3.44 (2.18)	**H(2) = 15.07, *p <* 0.001**^**c**^
						MCI-HC**
						MCI-SCD***
	**Primacy items**	2.74 (1.19)	2.78 (1.13)	3.01 (0.942)	1.92 (1.53)	**H(2) = 10.35, *p <* 0.01**^**c**^
						MCI-SCD**
	**Recency items**	2.67 (1.16)	2.94 (1.05)	2.89 (1.00)	1.72 (1.28)	**H(2) = 17.37, *p <* 0.001**^**c**^
						MCI-HC***
						MCI-SCD***
	**Serial clusters**	3.26 (2.40)	3.34 (2.59)	3.43 (2.17)	2.68 (2.75)	H(2) = 4.10, *p =* 0.13^c^

^a^Univariate ANOVA

^b^Chi-square test

^c^Kruskal–Wallis H test

^d^Mini–Mental State Examination

^*^p < 0.05, ^**^p < 0.01, ^***^p < 0.001, significant results are marked in bold.

### Group-level ICA-derived resting-state networks

Our ICA-derived RSNs showed moderate to strong spatial correlations with network templates from Shirer et al. [49], with correlation values of 0.77 for the DMN, 0.53 for the ECN, and 0.48 for the LAN. All selected components exhibited substantial overlap with key regions relevant to their respective networks.

**Fig. 2:**
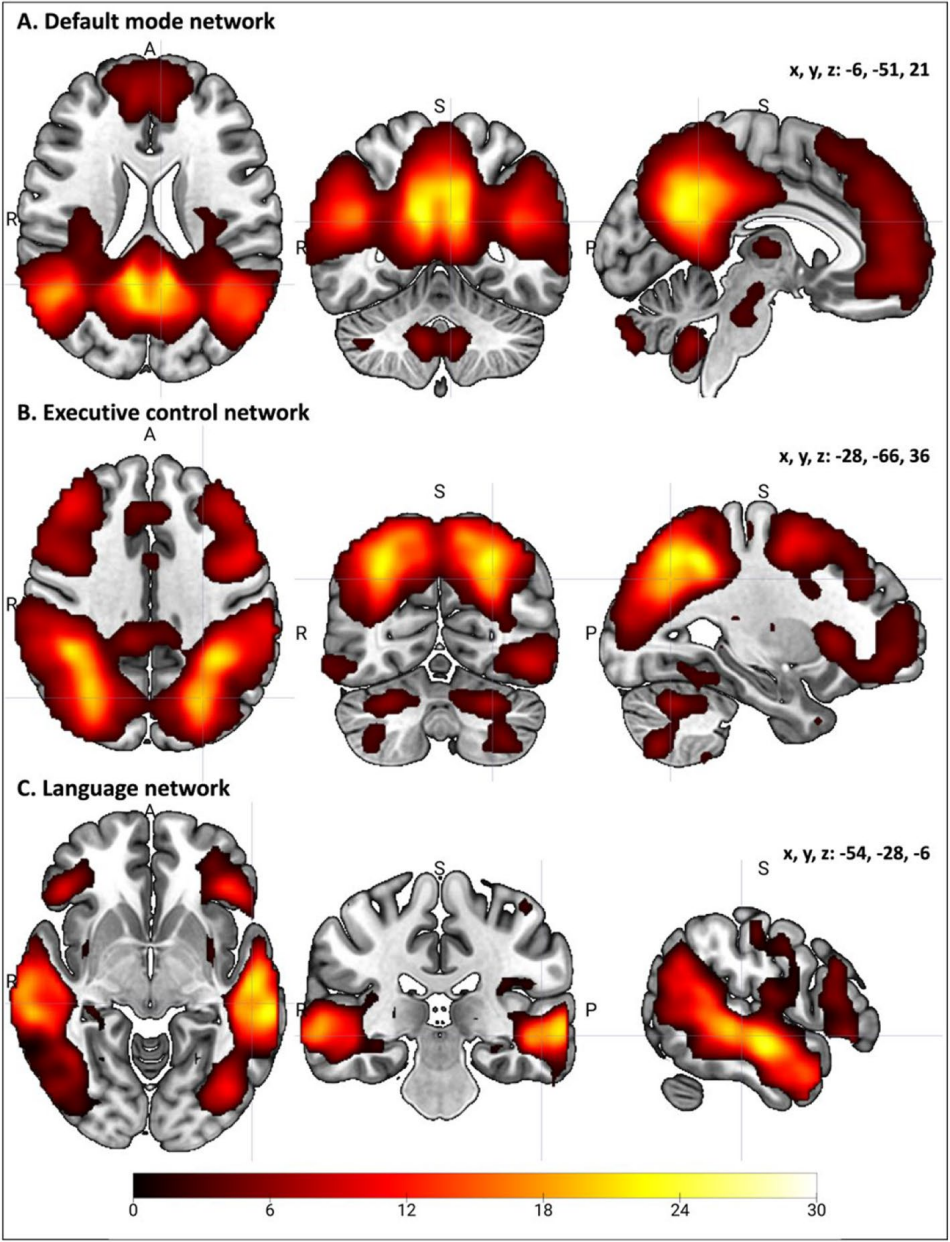
Group-level ICA-derived resting-state networks (RSNs). Spatial maps of the (**A**) default mode network (DMN), (**B**) executive control network (ECN), and (**C**) language network (LAN) identified using a forty-component ICA decomposition with an 8 mm smoothing kernel. DMN and ECN maps were visualized by displaying the top 10% of voxel intensities from the group-level component maps, whereas the LAN was thresholded at a peak T-value > 5, highlighting the most strongly contributing regions. These thresholded maps were also used to generate binarized masks for subsequent regression analyses. All results are shown in MNI space over a standard anatomical template. Images are shown in radiological convention (left = right hemisphere). The color scale represents *t*-values.

The DMN derived from ICA at the group-level was composed of bilateral regions and the spatial map was largely symmetric (Fig. 2A). Key regions included the precuneus, superior frontal gyrus, posterior orbitofrontal cortex, and ventral lateral thalamus. In the left hemisphere, clusters were observed in the cerebellum (Lobule 9 and Crus II) and inferior frontal gyrus (Table S2). The ECN showed strong connectivity in the left inferior parietal lobule and inferior temporal gyrus, with additional clusters in the right middle frontal gyrus and thalamus (Table S2, Fig. 2B). The LAN was primarily left-lateralized, with the largest cluster located in the left middle temporal gyrus. Additional clusters were found in the left precuneus and cerebellum (Lobule 8), and in the right cerebellum (Lobule 9) (Table S2, Fig. 2C).

Sensitivity analyses across multiple ICA configurations indicated that the DMN and ECN were highly stable. Group-level components captured most voxels from individual groups, with strong spatial correspondence across configurations (DMN Dice 0.74 ± 0.07, ECN Dice 0.75 ± 0.05; spatial correlation DMN 0.90 ± 0.06, ECN 0.88 ± 0.13; Table S3). The LAN was more variable (Dice 0.45 ± 0.07, spatial correlation 0.35 ± 0.22; Table S3), yet core regions such as left inferior frontal gyrus and left posterior superior temporal gyrus were consistently identified (Fig. S1).

### Network-specific resting-state connectivity associated with speech-based cognitive performance and features

**Fig. 3:**
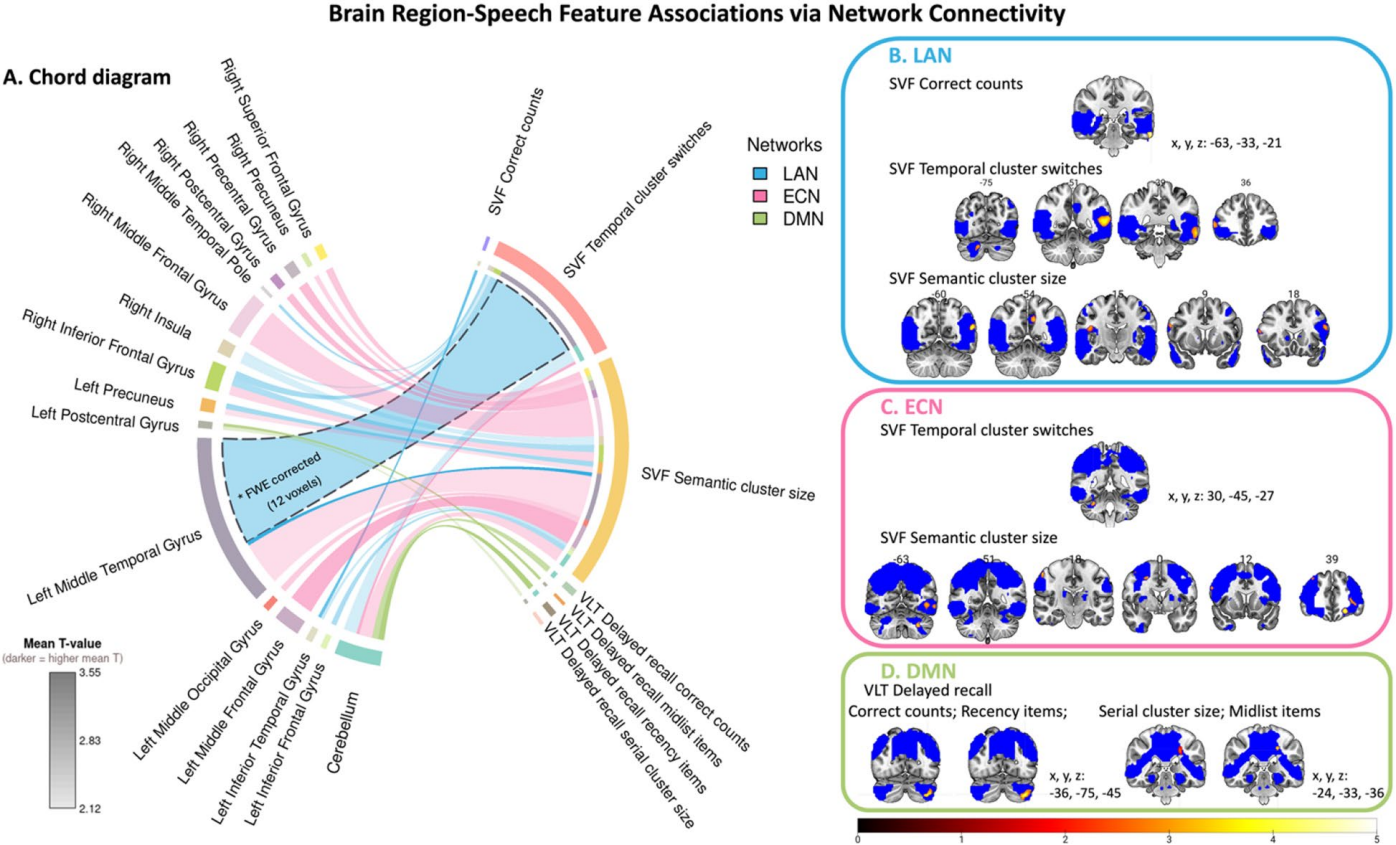
Brain Region-Speech Feature Relationships via Network Connectivity. Associations are shown at an uncorrected p < 0.01 with a minimum cluster size of 20 voxels. **(A) **Chord diagram illustrating associations between speech features and FC of brain regions within RSNs. Line thickness represents the number of significant voxels mapped to each network-level association, line transparency reflects the mean T-value across voxels (less transparent indicates stronger effects), and line color denotes the corresponding network. **(B–D) **Brain regions in which FC was associated with different speech features, mapped onto group-level RSN masks. Blue voxels indicate RSNs, and warm-colored voxels (T-value color scale) indicate regions showing significant associations. Statistical maps are overlaid on the MNI template brain. All models were adjusted for age, sex, education, diagnosis, scan site, and the time interval between speech assessment and MRI. Images are displayed in radiological convention (left = right hemisphere).

Of all the associations examined, the only statistically significant finding after correction was a positive association between LAN connectivity and SVF temporal cluster switching. This effect was localized to the left middle temporal gyrus (12 voxels, mean T = 3.86, peak T = 4.08; peak MNI coordinates: − 57, − 51, 12). Sensitivity analyses using an alternative ICA configuration confirmed that this association, along with other LAN-speech associations reported below, remained consistent across ICA template strategies (Table S4). To support interpretation, exploratory results using a liberal threshold (*p <* 0.01 uncorrected, cluster size ≥ 20 voxels) are also reported (Fig. 3, Table 2). All analyses were restricted to the group-level RSN masks.

**Table 2:** Brain regions showing significant associations between resting-state network connectivity and digital speech features

Digital speech features		network	Cluster size (voxels)	mean T	peak MNI Coordinates			peak T	Side	Anatomical location
SVF	Correct counts	LAN	23	3.08	−66	−33	−27	3.90	L	Inferior Temporal Gyrus
	Semantic cluster size	ECN	92	2.30	−30	−63	−39	3.81	L	Cerebellum Crus 1
			304	2.20	−42	−57	0	3.53	L	Middle Temporal Gyrus
			168	2.74	−24	39	−12	4.28	L	Middle Frontal Gyrus, part 2
			267	2.35	36	36	48	3.59	R	Middle Frontal Gyrus, part 2
			44	2.40	−24	−57	33	3.49	L	Middle Occipital Gyrus
			66	2.28	60	12	27	3.29	R	Opercular Inferior Frontal Gyrus
			35	2.30	18	−51	33	3.19	R	Precuneus
			54	2.46	21	0	45	3.49	R	Superior Frontal Gyrus, part 2
			42	2.15	0	−75	48	2.94	L	Precuneus
			53	2.60	54	−18	54	3.65	R	Postcentral Gyrus
			70	2.61	45	−9	54	3.59	R	Precentral Gyrus
		LAN	22	2.23	−57	−27	−18	2.75	L	Inferior Temporal Gyrus
			63	2.12	45	−15	21	3.29	R	Insula
			38	2.49	−54	18	21	3.32	L	Inferior Frontal Gyrus, triangular part
			51	2.47	57	9	24	3.94	R	Opercular Inferior Frontal Gyrus
			27	3.55	−57	−60	21	4.19	L	Middle Temporal Gyrus
			39	2.57	−12	−54	36	3.43	L	Precuneus
	Temporal cluster switches	ECN	21	2.77	30	−45	−27	3.38	R	Cerebellum Lobule 6
		LAN	94	2.14	12	−75	−33	3.18	R	Cerebellum Crus 2
			**733**	**2.52**	**−57**	**−51**	**12**	**4.08**	**L**	Middle Temporal Gyrus *
			21	2.58	57	12	−21	2.95	R	Middle Temporal Pole
			55	2.53	57	36	6	3.39	R	Inferior Frontal Gyrus, triangular part
			25	2.14	33	−9	9	3.05	R	Insula
VLT delayed recall	Correct counts	DMN	45	2.91	−36	−75	−45	4.08	L	Cerebellum Crus 2
	Midlist items	DMN	21	3.22	−24	−33	39	3.89	L	Postcentral Gyrus
	Recency items	DMN	42	3.36	−36	−75	−45	4.60	L	Cerebellum Crus 2
	Serial cluster size	DMN	24	2.32	−24	−33	36	2.74	L	Postcentral Gyrus

Uncorrected threshold of *p <*.01, with cluster size ≥ 20 voxels

^*^12 voxels in this cluster survived correction (mean T = 3.86, peak T = 4.08, p_FWE_ < 0.05), significant results are marked in bold.

#### Cognitive composite scores associated with connectivity in DMN, ECN, and LAN

No significant associations were observed between SB-C scores, either global or domain-specific, and connectivity in any of the three RSNs.

#### SVF features associated with connectivity in ECN and LAN

At an uncorrected threshold, SVF correct counts were associated with LAN connectivity in the left inferior temporal gyrus (Fig. 3, Table 2). SVF semantic cluster size showed a wide range of associations with ECN connectivity, involving the bilateral middle frontal gyri, left middle temporal gyrus, cerebellar regions, right inferior frontal and precentral areas, bilateral precuneus, and right superior and postcentral frontal cortices (Fig. 3, Table 2). Within the LAN, semantic cluster size was associated with connectivity in the left inferior and middle temporal gyri, bilateral inferior frontal gyrus, right insula, and left precuneus (Fig. 3, Table 2). Temporal cluster switching during SVF was associated with connectivity in the right cerebellum (Crus 2), left middle temporal gyrus, right middle temporal pole, right inferior frontal gyrus (triangular part), and right insular regions within the LAN (Fig. 3, Table 2), as well as the right cerebellar lobule VI within the ECN (Fig. 3, Table 2).

#### VLT delayed recall features associated with connectivity within DMN

Within the DMN, VLT delayed recall correct counts and recency items were associated with connectivity in the left cerebellar Crus II (Fig. 3, Table 2). Serial cluster size and midlist items were associated with connectivity in the left postcentral cortex (Fig. 3, Table 2).

#### Complementary analyses of speech-functional connectivity associations

Models including speech feature × diagnosis interaction terms revealed limited evidence for group-specific associations. While some voxel-wise interactions survived TFCE correction, primarily within LAN-related regions, the majority of these significant voxels fell outside our predefined IC network masks. The only interaction effect that localized within our RSNs was confined to 3 voxels in the ECN. These findings suggest that the associations between speech-derived measures and functional connectivity do not differ substantially across diagnostic groups at the network level (Table S6).

Seed-to-voxel analyses, which complement the ICA-based network findings, are provided in the Supplementary Materials. The spatial patterns of the seed-defined RSNs largely aligned with ICs, supporting the consistency of network identification across methods (Fig. S2). However, associations with digital speech-based metrics showed partial overlap, indicating method-dependent variability in regression sensitivity. Importantly, these seed-based associations were exploratory, identified using an uncorrected threshold (*p <* 0.01, cluster size ≥ 20 voxels, Table S7, Fig. S2).

## Discussion

This study investigated associations between digital speech–derived cognitive composite scores, task-specific speech features, and rsFC within the DMN, ECN, and LAN across 129 individuals from cognitively healthy to MCI in the German PROSPECT-AD study. We hypothesized that both SB-C scores and speech features would be sensitive to subtle network-specific FC alterations in at-risk populations.

### Primary finding: temporal cluster switching and FC in LAN

The significant result was that stronger LAN connectivity was associated with greater temporal cluster switching during SVF (TFCE-based FWE *p <* 0.05), suggesting more dynamic lexical access. This association was mostly observed in the left middle temporal gyrus, a region that has been implicated both in goal-directed and automatic semantic processes, as well as in controlled semantic retrieval [55]. These cognitive processes align with what the temporal switching feature captures: how verbal responses are arranged over time and the flexible retrieval strategies used during the task. Previous analyses combining temporal and semantic features have shown that these verbal patterns tend to decline in individuals with MCI and AD, even when semantic knowledge remains relatively preserved [41]. Our results are consistent with this theoretical relationship, indicating that temporal speech dynamics should be given priority in future research as a promising speech indicator of early LAN changes. However, the modest effect size and limited spatial extent require cautious interpretation. Replication in independent samples will be essential to confirm whether temporal cluster switching can reliably track LAN integrity among at-risk populations of AD.

### Absence of associations with SB-C scores

Our ICA-based rsFC analyses did not find any significant association with global or domain-specific SB-C scores. Composite cognitive measures tend to have greater reliability and sensitivity than individual test scores in particular cognitive domains. They are therefore recommended for identifying subtle cognitive changes in at-risk groups [56, 57]. Additionally, common composites such as the Cognitive Function Composite (CFC) are less affected by practice effects, which makes them a suitable endpoint in longitudinal studies or clinical trials [2, 57]. Despite these theoretical advantages, the absence of significant findings contrasts with previous studies linking cognitive composites to rsFC in MCI [30, 58] and with our own prior PROSPECT-AD findings showing SB-C associations with clinical measures and brain atrophy across diagnostic groups [16, 17].

One explanation could be the intended purpose of SB-C scores. SB-C scores have been designed for assessing general cognitive performance across executive function, memory, and processing speed [39]. While our objective was to examine the broader clinical potential of SB-C scores by linking them to underlying brain FC alterations, this inclusivity may fundamentally limit their sensitivity to network-specific FC changes, highlighting a general challenge of using composite scores to detect subtle neuropathology. One real-world example is the Preclinical Alzheimer's Cognitive Composite (PACC). Although PACC is widely used to track cognitive decline in preclinical AD [59], studies have indicated varying degrees of reliability in detecting baseline Aβ status [60, 61] . This observation underscores that general composite scores have limitations in certain contexts. Also, the subtle nature of network FC alterations at very early stages of AD may exceed the detection threshold of broad composite measures. This suggests that composite cognitive measures might require support from complementary approaches to capture network-specific brain alterations more sensitively.

Rather than relying solely on composite scores that aggregate across cognitive domains, future research could use data-driven techniques such as principal component analysis of speech features to apply more analytical strategies focused on the speech-FC connection. By utilizing both the clinical interpretability of domain-level scores and the discovery potential of unsupervised methods, this dual strategy could potentially uncover speech features that are more closely linked to underlying FC patterns.

### Methodological considerations: ICA approach and network identification

Initial analyses (uncorrected *p <* 0.01) suggested additional connections between SVF features and ECN/LAN connectivity, and between VLT delayed recall features and DMN connectivity. We therefore systematically evaluated whether our ICA approach introduced bias that could account for the absence of significant associations between rsFC and SB-C scores, as well as the largely exploratory nature of the rsFC-speech findings. We raised this concern because heterogeneity in automated RSN derivation remains a recognized methodological challenge, as analytical parameter variations can substantially alter network topology and localization [47, 48].

Spatial maps of the DMN and ECN were highly consistent across all ICA pooling configurations, with balanced contributions from diagnostic groups. This ruled out meaningful bias from our SCD-dominant sample (56% of participants). In contrast, LAN identification proved more challenging. Across all configurations, 40 components were required to isolate a single network covering all key language-related regions, with no clear hemispheric subdivision. This contrasts with previous work using substantially larger samples, which identified left- and right-lateralized language-related networks applying only 20 components [50]. The LANs we identified also showed greater spatial variability across ICA configurations.

This pattern is in line with heterogeneous LAN alterations in the AD spectrum reported previously, including reduced connectivity within semantic control networks in mild AD [32] and increased LAN activations in MCI [62]. However, when we examined individual spatial FC maps, we did not find significant differences between diagnostic groups. This may reflect the early-stage expression of LAN alterations at the pre-dementia stages represented in our sample. This interpretation is consistent with prior DELCODE findings where LAN showed only chance-level classification performance in SCD and MCI, with group differences emerging only under liberal thresholds [23, 26].

When we reran analyses using a different LAN template, LAN rsFC-speech associations remained highly consistent, indicating that the ICA pooling approach did not materially influence our results. Instead, this pattern is more plausibly attributable to the small effect sizes characteristic of rsFC–speech associations in heterogeneous early-stage AD populations, consistent with large-scale brain-wide association studies showing that relationships between complex behavioral phenotypes and FC are much weaker than previously assumed, requiring sample sizes in the thousands for reliable detection [63]. Given our present sample size, limited statistical power rather than methodological bias or absent brain-behavior relationships likely accounts for the observed results.

### Complementary seed-based analyses

We performed seed-based analyses to supplement our ICA results. The reliability of our ICA-derived RSN characterization was supported by consistent network identification between the two methods. Seed analyses also revealed associations between speech features and FC at the exploratory level. However, we do not recommend relying primarily on seed-based approaches or applying them as an exclusive analytical strategy. Meta-analyses have demonstrated that even minor changes in seed selection within the DMN can produce substantial variability in results, raising questions about the reliability of such methods [64]. Employing multiple analytical strategies may therefore yield complementary insights. Replication in independent populations, in addition to testing alternative implementations of analytic approaches, may enable future studies to reach credible and generalizable results while avoiding spurious associations.

### Study strengths and limitations

While most remote cognitive assessment research has focused on group classification, cognitive associations, or amyloid burden correlations, we extended this by exploring links between SB-C scores, speech features, and brain functional connectivity to better understand their neural correlates. This hypothesis-generating study provides important methodological and conceptual insights for future research, but it also has limitations.

The one-year average interval between rs-fMRI and speech assessments is a crucial limitation. Although this has been statistically controlled, it may still add variability that limits precision. This limitation cannot be addressed within the current dataset, as data collection is already complete. However, it will be investigated in forthcoming longitudinal analyses that focus on associations between FC and speech changes over time. Another limitation of our study could also be that the methodologies that were focused on global FC may have potentially missed local connectivity. Hence, our findings might be complemented by using local connectivity metrics such as amplitude of low-frequency fluctuations (ALFF) or regional homogeneity (ReHo).

## Conclusions

This study provides proof-of-concept that specific speech features, particularly temporal dynamics during SVF, show detectable associations with FC in LAN, even in early-stage populations spanning from cognitively healthy individuals to MCI. However, the effects are modest in size and limited in spatial extent, requiring replication before clinical application. Notably, SB-C scores did not show sufficient sensitivity to capture network-specific brain FC changes in our cohort, although they remain useful for tracking brain volumetric changes and general cognitive decline.

Future studies should use longitudinal designs that track rsFC-speech associations over time, significantly increase sample sizes to achieve an adequate statistical power, and harmonize data collection procedures across research sites while validating findings in diverse cohorts across different clinical stages and sociodemographic backgrounds in order to advance the development of speech-based digital tools for identifying and monitoring brain function alterations in populations at-risk of AD.

## Supplementary Information

Supplementary Material 1.

## Data Availability

The dataset(s) supporting the conclusions of this article are not publicly available due to institutional policy, but are available from the corresponding author on reasonable request.

## Abbreviations

AD: Alzheimer’s disease
MCI: Mild cognitive impairment
SVF: Semantic Verbal Fluency
VLT: Verbal Learning Task
SCD: Subjective cognitive decline
Aβ: Amyloid beta
SB-C score: Speech-based cognitive score
FC: Functional connectivity
rs-fMRI: Resting-state functional MRI
DMN: Default mode network
ECN: Executive control network
LAN: Language network
HC: Healthy controls
rsFC: Resting-state functional connectivity
ICA: Independent component analysis
IC: Independent component
RSN: Resting-state network
TFCE: Threshold-Free Cluster Enhancement
FWE: Family-wise error
