# Supplementary material for: Exploring neural correlates of automated speech-based cognitive markers through resting-state functional connectivity in aging and at-risk Alzheimer’s disease
Source: Alzheimers Res Ther. 2026 Feb 24;18:52. doi: 10.1186/s13195-026-01993-x (PMC12961854; doi:10.1186/s13195-026-01993-x)
Supplement: Supplementary file 1 — Supplementary Material 1. [file 13195_2026_1993_MOESM1_ESM.docx]

## **Table S1**Overview of selected speech features

| **Category** | **Features** | **Description** |
| --- | --- | --- |
| SVF features | Correct count | The total number of animal names correctly produced in one minute. |
|  | Word frequency | Average frequency of produced animal names based on a German corpus. |
|  | Semantic cluster size | Average number of semantically related words produced consecutively in a subcategory (e.g., farm animals). |
|  | Semantic cluster switches | The total number of switches between semantic subcategories (n_clusters - 1). |
|  | Temporal cluster size | Average size of word groups produced in rapid succession (based on pauses). |
|  | Temporal cluster switches | Number of transitions between temporal clusters (n_clusters - 1). |
| VLT delayed recall features | Delayed recall count | Number of correctly remembered words in the delayed recall trial. |
|  | Serial clusters | The number of instances where two or more words originally presented consecutively are recalled in the correct order. |
|  | Primacy items | The number of words correctly recalled that belong to the first five words in learning trials. |
|  | Midlist items | The number of words correctly recalled that belong to the middle five words (words 6 to 10) in learning trials. |
|  | Recency items | The number of words correctly recalled that belong to the last five words in learning trials. |

## **Table S2** Location of functional connectivity for ICA-driven RSNs of interest

| **Network** | **Cluster size (voxels)** | **mean T** | **peak MNI Coordinates** | | | **peak T** | **Side** | **Anatomical location** |
| --- | --- | --- | --- | --- | --- | --- | --- | --- |
|  |  |  |  |  |  |  |  |  |
| **DMN** | 285 | 3.78 | -6 | -54 | -42 | 7.15 | L | Cerebellum Lobule 9 |
|  | 1189 | 3.47 | -12 | -87 | -39 | 6.53 | L | Cerebellum Crus II |
|  | 13118 | 6.82 | 9 | -54 | 24 | 24.41 | R | Precuneus |
|  | 5389 | 4.59 | 21 | 30 | 45 | 10.80 | R | Superior Frontal Gyrus |
|  | 86 | 2.65 | 33 | 21 | -15 | 3.55 | R | Posterior Orbitofrontal Cortex |
|  | 126 | 3.04 | 12 | -9 | 6 | 4.57 | R | Ventral Lateral Thalamus |
|  | 37 | 2.60 | -54 | 24 | 9 | 3.11 | L | Inferior Frontal Gyrus |
| **ECN** | 12182 | 7.44 | -36 | -48 | 45 | 26.69 | L | Inferior Parietal Lobule |
|  | 167 | 2.69 | 33 | 0 | -51 | 3.90 | L | Inferior Temporal Gyrus |
|  | 8046 | 5.20 | 42 | 39 | 30 | 13.57 | R | Middle Frontal Gyrus |
|  | 168 | 2.66 | 21 | -27 | 12 | 3.88 | R | Thalamus |
| **LAN** | 284 | 6.38 | 18 | -81 | -42 | 9.10 | R | Cerebellum Crus II |
|  | 5534 | 8.79 | -63 | -33 | -3 | 21.33 | L | Middle Temporal Gyrus |
|  | 62 | 6.28 | -21 | -84 | -36 | 8.76 | L | Cerebellum Crus II |
|  | 5022 | 8.15 | 63 | -33 | -6 | 18.42 | R | Middle Temporal Gyrus |
|  | 65 | 5.77 | 0 | 54 | -12 | 7.08 | L | Medial Orbitofrontal Cortex |
|  | 76 | 5.65 | -24 | -36 | 0 | 7.15 | L | Hippocampus |
|  | 116 | 5.93 | -6 | 51 | 27 | 7.85 | L | Medial Superior Frontal Gyrus |
|  | 20 | 5.46 | 24 | -75 | 24 | 6.14 | R | Precuneus |
|  | 82 | 6.17 | -6 | -51 | 36 | 7.72 | L | Precuneus |
|  | 96 | 5.88 | -36 | 6 | 45 | 7.41 | L | Middle Frontal Gyrus |

DMN and ECN maps were thresholded at the top 10% of voxel intensities from the group-level component maps, whereas the LAN was thresholded at a peak T-value > 5, with cluster size ≥ 20 voxels.

# **Supplement 1.** Spatial consistency of resting-state networks across diagnostic groups

*ICA spatial maps comparison*

To address concerns about potential bias from pooling diagnostic groups, we conducted targeted sensitivity analyses comparing the original pooled template (ALL, n=129: 32 HC, 72 SCD, 25 MCI) with three alternative configurations: (1) separate ICAs performed independently within each diagnostic group, (2) a balanced pooling approach using a subsampled SCD group (BALANCE, n=93: 32 HC, 36 SCD, 25 MCI), and (3) an HC+MCI pooling approach excluding the SCD group entirely (HCMCI, n=57: 32 HC, 25 MCI). These configurations test whether disease-specific spatial characteristics are diluted by pooling and whether the SCD-dominant sample biases template derivation. We identified DMN, ECN, and LAN across all configurations and calculated spatial correspondence using Dice coefficients, Jaccard indices, and spatial correlations while applying consistent thresholding. Results are presented in Table S3 and Fig. S1.

Given that the LAN showed greater spatial variability across configurations compared to DMN and ECN, we reran all statistical models using the balanced-sample LAN template to verify that our primary findings were not artifacts of the pooling strategy. These sensitivity analyses are reported in Table S4.

## **Fig. S1** Spatial comparison of RSNs across different ICA configurations


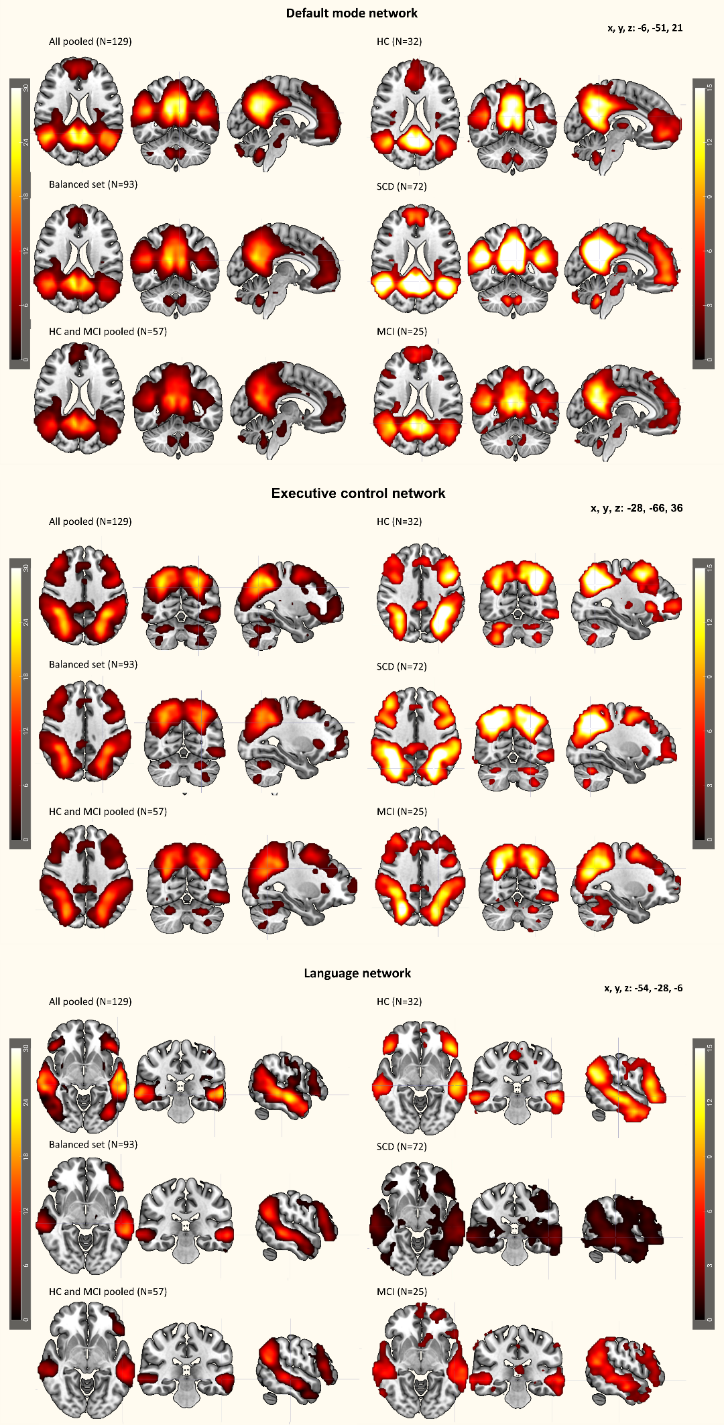


Spatial maps of the Default Mode Network (top), Executive Control Network (middle), and Language Network (bottom) derived from different ICA pooling strategies. Each row shows results from: All pooled (N=129), Balanced set (N=93), HC and MCI pooled (N=57), HC only (N=32), SCD only (N=72), and MCI only (N=25). Color scale represents component weights (coordinates indicate slice positions). DMN and ECN maps were visualized by displaying the top 10% of voxel intensities from the group-level component maps, whereas the LAN was thresholded at a peak T-value > 5.

## **Table S3** Quantitative comparison of network spatial correspondence across ICA pooling strategies

| **Analysis based on RSN maps already thresholded** | | | | | | |
| --- | --- | --- | --- | --- | --- | --- |
| Voxel size: 3 x 3 x 3 mm | | | | | | |
| **Overlapped with all pooled ICA-derived resting-state networks** | | | | | | |
| Network | Group | Dice | Jaccard | SpatCorr | Ovlp_G->P | COM_dist |
| DMN | HC | 0.683 | 0.518 | 0.858 | 76.89 | 1.61 |
|  | MCI | 0.716 | 0.558 | 0.871 | 78.10 | 2.71 |
|  | SCD | 0.845 | 0.737 | 0.979 | 87.30 | 2.21 |
|  | BALANCE | 0.764 | 0.618 | 0.926 | 84.35 | 6.36 |
|  | HCMCI | 0.685 | 0.521 | 0.843 | 77.53 | 8.30 |
| ECN | HC | 0.672 | 0.506 | 0.652 | 75.61 | 16.95 |
|  | MCI | 0.742 | 0.589 | 0.881 | 81.85 | 3.95 |
|  | SCD | 0.796 | 0.661 | 0.957 | 85.61 | 3.94 |
|  | BALANCE | 0.772 | 0.629 | 0.975 | 87.01 | 3.73 |
|  | HCMCI | 0.771 | 0.627 | 0.929 | 85.56 | 4.70 |
| LAN | HC | 0.428 | 0.272 | 0.133 | 36.56 | 30.39 |
|  | MCI | 0.395 | 0.246 | 0.383 | 36.95 | 22.76 |
|  | SCD | 0.568 | 0.397 | 0.698 | 49.69 | 22.72 |
|  | BALANCE | 0.460 | 0.299 | 0.379 | 55.02 | 26.57 |
|  | HCMCI | 0.408 | 0.256 | 0.174 | 45.43 | 31.31 |
| **Network-specific summary** | | | | | | |
| DMN | Dice | | | 0.74 ± 0.07 | [0.68 - 0.85] | |
|  | Jaccard | | | 0.59 ± 0.09 | [0.52 - 0.73] | |
|  | Spatial Corr | | | 0.90 ± 0.06 | [0.84 - 0.98] | |
| ECN | Dice | | | 0.75 ± 0.05 | [0.67 - 0.80] | |
|  | Jaccard | | | 0.60 ± 0.06 | [0.51 - 0.66] | |
|  | Spatial Corr | | | 0.88 ± 0.13 | [0.65 - 0.98] | |
| LAN | Dice | | | 0.45 ± 0.07 | [0.40 - 0.57] | |
|  | Jaccard | | | 0.29 ± 0.06 | [0.25 - 0.40] | |
|  | Spatial Corr | | | 0.35 ± 0.22 | [0.13 - 0.70] | |
| **Overlapped with BALANCE ICA-derived resting-state networks** | | | | | | |
| Network | Group | Dice | Jaccard | SpatCorr | Ovlp_G->P | COM_dist |
| DMN | HC | 0.742 | 0.589 | 0.901 | 75.52 | 4.78 |
|  | MCI | 0.769 | 0.625 | 0.913 | 76.06 | 6.50 |
|  | SCD | 0.761 | 0.614 | 0.902 | 71.65 | 6.21 |
|  | ALL | 0.764 | 0.618 | 0.926 | 69.83 | 6.36 |
|  | HCMCI | 0.820 | 0.695 | 0.946 | 83.88 | 3.91 |
| ECN | HC | 0.717 | 0.559 | 0.620 | 71.59 | 17.97 |
|  | MCI | 0.822 | 0.698 | 0.862 | 80.71 | 1.58 |
|  | SCD | 0.869 | 0.769 | 0.939 | 83.35 | 0.66 |
|  | ALL | 0.772 | 0.629 | 0.975 | 69.42 | 3.73 |
|  | HCMCI | 0.850 | 0.739 | 0.896 | 83.88 | 3.43 |
| LAN | HC | 0.693 | 0.530 | 0.437 | 68.14 | 16.10 |
|  | MCI | 0.592 | 0.421 | 0.565 | 64.73 | 3.05 |
|  | SCD | 0.647 | 0.478 | 0.699 | 65.47 | 10.45 |
|  | ALL | 0.483 | 0.318 | 0.503 | 37.32 | 20.58 |
|  | HCMCI | 0.779 | 0.638 | 0.764 | 76.72 | 7.35 |
| **Network-specific summary** | | | | | | |
| DMN | Dice | | | 0.78 ± 0.03 | [0.74 - 0.82] | |
|  | Jaccard | | | 0.64 ± 0.04 | [0.59 - 0.70] | |
|  | Spatial Corr | | | 0.92 ± 0.02 | [0.90 - 0.95] | |
| ECN | Dice | | | 0.82 ± 0.06 | [0.72 - 0.87] | |
|  | Jaccard | | | 0.69 ± 0.08 | [0.56 - 0.77] | |
|  | Spatial Corr | | | 0.87 ± 0.13 | [0.62 - 0.98] | |
| LAN | Dice | | | 0.65 ± 0.10 | [0.48 - 0.78] | |
|  | Jaccard | | | 0.49 ± 0.11 | [0.32 - 0.64] | |
|  | Spatial Corr | | | 0.62 ± 0.14 | [0.44 - 0.76] | |

Dice: Dice coefficient (spatial overlap)

Jaccard: Jaccard index (intersection/union)

SpatCorr: Spatial correlation of intensities

Ovlp_G->P: % of group voxels found in pooled map

COM_dist: Euclidean distance between centers of mass (mm)

## **Table S4** Brain regions showing significant associations between LAN connectivity and digital speech features using a balanced template

| **Digital speech features** | | **network** | **Cluster size (voxels)** | **mean T** | **peak MNI Coordinates** | | | **peak T** | **Side** | **Anatomical location** |  |
| --- | --- | --- | --- | --- | --- | --- | --- | --- | --- | --- | --- |
|  |  |  |  |  |  |  |  |  |  |  |  |
| SVF | Correct counts | LAN | 23 | 2.71 | -60 | -36 | -18 | 3.38 | L | Inferior Temporal Gyrus |  |
|  |  |  | 76 | 2.67 | -66 | -24 | -6 | 3.46 | L | Middle Temporal Gyrus |  |
|  |  |  | 26 | 2.62 | -39 | 42 | -3 | 3.29 | L | Inferior frontal gyrus |  |
|  | Word frequency | LAN | 34 | -2.78 | -66 | -21 | -3 | -3.28 | L | Middle Temporal Gyrus |  |
|  | Semantic cluster size | LAN | 289 | 2.86 | -60 | -12 | -12 | 4.29 | L | Middle Temporal Gyrus |  |
|  | Temporal cluster switches | LAN | 25 | 2.10 | 36 | -75 | -48 | 2.79 | R | Cerebellum Crus 2 |  |
|  |  |  | 29 | 2.28 | -63 | -9 | -24 | 2.73 | L | Middle Temporal Gyrus |  |
|  |  |  | 504 | 2.56 | -57 | -51 | 9 | 3.60 | L | Middle Temporal Gyrus |  |
|  |  |  | 21 | 2.25 | 51 | -39 | -6 | 2.89 | R | Middle Temporal Gyrus |  |
|  |  |  | 52 | 2.35 | 72 | -42 | 6 | 3.69 | R | Middle Temporal Gyrus |  |
|  |  |  | 151 | 2.32 | -54 | 36 | 12 | 3.28 | L | Inferior Frontal Gyrus, triangular part |  |
|  |  |  | 39 | 2.32 | 57 | -51 | 18 | 3.74 | R | Middle Temporal Gyrus |  |

Uncorrected threshold of p < .01, with cluster size ≥ 20 voxels.

*Individual spatial maps comparison*

To assess whether the intrinsic connectivity networks (DMN, ECN, LAN) derived from the pooled ICA were spatially consistent across diagnostic groups, we performed dual regression followed by voxel-wise non-parametric permutation testing (FSL randomize, 5,000 permutations). We tested for differences in all directions (e.g., HC > MCI and HC < MCI). As summarized in Table S5, no significant spatial differences survived TFCE correction all corrected p > 0.05), indicating that the functional architecture of these networks remained spatially stable across the AD-risk spectrum in this cohort.

## **Table S5** Statistical comparisons for spatial differences in network maps (DMN, ECN, LAN) between diagnostic groups

| **Comparison (Direction)** | **DMN** | | | **ECN** | | | **LAN** |  |  |
| --- | --- | --- | --- | --- | --- | --- | --- | --- | --- |
|  | peak T | p_uncorr_ | p_FWE_ | peak T | p_uncorr_ | p_FWE_ | peak T | p_uncorr_ | p_FWE_ |
| HC < SCD | 4.32 | 0.001 | 0.819 | 4.27 | 0.046 | 1.00 | 4.51 | 0.009 | 0.967 |
| HC > SCD | 4.49 | 0.023 | 0.998 | 4.00 | 0.061 | 1.00 | 4.00 | 0.014 | 0.969 |
| HC < MCI | 3.86 | 0.007 | 0.961 | 3.98 | 0.047 | 0.996 | 4.10 | 0.012 | 0.994 |
| HC > MCI | 3.95 | 0.024 | 0.995 | 3.81 | 0.004 | 0.916 | 4.14 | 0.004 | 0.963 |
| SCD > MCI | 4.83 | <0.001 | 0.425 | 4.12 | <0.001 | 0.450 | 4.60 | <0.001 | 0.389 |
| SCD < MCI | 4.29 | <0.001 | 0.428 | 4.64 | <0.001 | 0.530 | 4.21 | <0.001 | 0.431 |

# **Supplement 2.** Seed-based functional connectivity analysis

## Imaging processing

After normalization, preprocessing included regression of nuisance covariates, white matter, cerebrospinal fluid (CSF), global signal, and 24 head motion parameters, from the fMRI time series. Global signal regression was included as a denoising step to reduce global noise across voxels, thereby enhancing the specificity of connectivity estimates, particularly in seed-based approaches (1). The residual time series were then band-pass filtered (0.01–0.1 Hz) and spatially smoothed using a 4 mm FWHM Gaussian kernel.

## Seed region definition

Seed regions of interest (ROIs) were defined based on prior literature and meta-analytic findings. To investigate connectivity within the DMN, we selected two spherical seeds: the right posterior cingulate cortex (rPCC; MNI: 4, -52, 28) and the left medial prefrontal cortex (mPFC; MNI: -2, 50, 6), based on meta-analytic coordinates (2). For examining LAN, seeds were placed in the left inferior frontal gyrus (IFG; MNI: -54, 24, 3) and the left posterior middle temporal gyrus (pMTG; MNI: -66, -42, 3), following prior studies (3). To assess connectivity within ECN, we employed two subdivisions of the dorsolateral prefrontal cortex (DLPFC): an anterior-ventral cluster (anPFC; center of gravity: MNI 30, 43, 23) and a posterior-dorsal cluster (dlPFC; MNI: 37, 33, 32), as stated in a previous study (4). All spherical seeds were defined with a 6 mm radius, centered on their respective MNI coordinates.

## Resting-state functional connectivity map generation

For each subject, RSFC maps were generated using DPARSFA by computing Pearson correlations between the mean time series of each seed region and the time series of all other gray matter voxels. The resulting correlation maps were then transformed to Fisher's z-scores to improve normality. Group-level RSFC maps were generated in SPM12 using one-sample t-tests across subjects (no covariates included), thresholded at family-wise error (FWE) corrected p < 0.05, with a minimum cluster size of 20 voxels. The resulting significant clusters were binarized to create seed-specific network masks, which were subsequently used to constrain the voxel space in multiple regression analyses.

## Statistical analysis

Second-level analyses were conducted using multiple regression models in SPM12 to examine the associations of RSFC maps with speech-derived composite cognitive (SB-C) scores and task-specific speech features, separately. Age, sex, years of education, diagnosis, and scan site were included as covariates. For each analysis, contrasts were defined by assigning a weight of 1 to the factor of interest, except for SVF word frequency, where a contrast weight of -1 was used to test for negative associations.

Statistical significance was determined using FWE correction at p < 0.05. When no clusters survived FWE correction, an exploratory threshold of uncorrected p < 0.01 with a minimum cluster size of 20 voxels was applied. Significant results were constrained within group-level RSFC network masks and visualized using *MRIcroGL.*

## Reference

1. Yan CG, Cheung B, Kelly C, Colcombe S, Craddock RC, Di Martino A, et al. A comprehensive assessment of regional variation in the impact of head micromovements on functional connectomics. NeuroImage. 2013 Aug;76:183–201.

2. Wang Y, Li Q, Yao L, He N, Tang Y, Chen L, et al. Shared and differing functional connectivity abnormalities of the default mode network in mild cognitive impairment and Alzheimer’s disease. Cereb Cortex [Internet]. 2024 Mar 1 [cited 2025 July 9];34(3). Available from: https://academic.oup.com/cercor/article/doi/10.1093/cercor/bhae094/7634124

3. Montembeault M, Chapleau M, Jarret J, Boukadi M, Laforce R, Wilson MA, et al. Differential language network functional connectivity alterations in Alzheimer’s disease and the semantic variant of primary progressive aphasia. Cortex. 2019 Aug;117:284–98.

4. Cieslik EC, Zilles K, Caspers S, Roski C, Kellermann TS, Jakobs O, et al. Is There “One” DLPFC in Cognitive Action Control? Evidence for Heterogeneity From Co-Activation-Based Parcellation. Cereb Cortex. 2013 Nov;23(11):2677–89.

## **Table S6**Diagnostic group differences in associations between RSN connectivity and speech-derived cognitive features

| **Speech features** | **Comparison (Direction)** | **DMN** | | | **ECN** | | | **LAN** | | |
| --- | --- | --- | --- | --- | --- | --- | --- | --- | --- | --- |
|  |  | peak T | p_uncorr_ | p_FWE_ | peak T | p_uncorr_ | p_FWE_ | peak T | p_uncorr_ | p_FWE_ |
| SB-C global score | HC<SCD | 4.10 | 0.007 | 0.942 | 4.15 | 0.004 | 0.632 | 4.40 | 0.004 | 0.738 |
|  | HC>SCD | 4.67 | 0.008 | 0.789 | 5.07 | 0.004 | 0.232 | 4.67 | 0.002 | 0.478 |
|  | HC<MCI | 3.82 | 0.024 | 0.932 | 4.50 | 0.034 | 0.987 | 4.11 | 0.062 | 0.994 |
|  | HC>MCI | 3.58 | 0.046 | 0.993 | 4.36 | 0.024 | 0.974 | 3.88 | 0.033 | 0.947 |
|  | SCD>MCI | 4.11 | 0.003 | 0.627 | 4.59 | 0.005 | 0.667 | 4.60 | 0.008 | 0.576 |
|  | SCD<MCI | 5.05 | **<0.001** | 0.244 | 4.17 | **<0.001** | 0.429 | 4.94 | **<0.001** | 0.443 |
| SB-C executive score | HC<SCD | 4.37 | 0.004 | 0.798 | 3.82 | 0.0382 | 0.998 | 4.04 | 0.032 | 0.959 |
|  | HC>SCD | 4.40 | 0.003 | 0.393 | 4.43 | 0.094 | 0.999 | 3.99 | 0.037 | 0.994 |
|  | HC<MCI | 6.02 | **<0.001** | 0.208 | 5.04 | 0.003 | 0.328 | 6.09 | **<0.001** | **0.034** |
|  | HC>MCI | 4.82 | **<0.001** | 0.249 | 4.31 | 0.045 | 0.969 | 5.98 | **<0.001** | 0.232 |
|  | SCD>MCI | 6.95 | **<0.001** | 0.096 | 5.50 | **<0.001** | 0.388 | 6.84 | **<0.001** | **0.043** |
|  | SCD<MCI | 5.28 | **<0.001** | 0.379 | 4.33 | 0.007 | 0.656 | 6.92 | **<0.001** | 0.055 |
| SB-C memory score | HC<SCD | 4.20 | 0.005 | 0.952 | 4.22 | 0.007 | 0.744 | 4.46 | 0.002 | 0.954 |
|  | HC>SCD | 4.10 | 0.042 | 0.995 | 5.28 | **<0.001** | 0.876 | 4.40 | **<0.001** | 0.820 |
|  | HC<MaCI | 3.90 | 0.080 | 0.968 | 4.88 | 0.008 | 0.865 | 3.51 | 0.0478 | 0.996 |
|  | HC>MCI | 4.11 | 0.058 | 0.948 | 4.00 | 0.006 | 0.831 | 3.92 | **<0.001** | 0.378 |
|  | SCD>MCI | 4.41 | **<0.001** | 0.788 | 4.25 | 0.004 | 0.609 | 4.93 | **<0.001** | 0.666 |
|  | SCD<MCI | 4.07 | **<0.001** | 0.529 | 4.78 | 0.008 | 0.536 | 4.67 | **<0.001** | 0.366 |
| SB-C processing speed score | HC<SCD | 4.17 | 0.007 | 0.890 | 4.90 | **<0.001** | 0.263 | 5.35 | **<0.001** | 0.508 |
|  | HC>SCD | 4.31 | 0.004 | 0.880 | 6.01 | **<0.001** | **0.033** | 4.86 | 0.002 | 0.493 |
|  | HC<MCI | 3.61 | 0.028 | 0.988 | 4.77 | 0.020 | 0.937 | 4.69 | **<0.001** | 0.758 |
|  | HC>MCI | 4.11 | 0.009 | 0.966 | 4.81 | 0.044 | 0.865 | 3.72 | 0.07 | 0.837 |
|  | SCD>MCI | 4.32 | **<0.001** | 0.528 | 4.92 | **<0.001** | 0.294 | 5.24 | **<0.001** | 0.384 |
|  | SCD<MCI | 5.05 | **<0.001** | 0.560 | 5.02 | **<0.001** | 0.244 | 5.57 | **<0.001** | 0.794 |
| SVF correct count | HC<SCD | - | - | - | 3.96 | 0.088 | 0.872 | 4.08 | 0.080 | 0.924 |
|  | HC>SCD | - | - | - | 3.91 | 0.038 | 0.979 | 4.05 | 0.008 | 0.888 |
|  | HC<MCI | - | - | - | 6.22 | **<0.001** | 0.088 | 7.68 | **<0.001** | **0.040** |
|  | HC>MCI | - | - | - | 4.26 | 0.034 | 0.806 | 7.38 | **<0.001** | **0.048** |
|  | SCD>MCI | - | - | - | 6.97 | **<0.001** | 0.073 | 8.78 | **<0.001** | **0.020** |
|  | SCD<MCI | - | - | - | 5.53 | **<0.001** | 0.333 | 8.30 | **<0.001** | **0.037** |
| SVF word frequency | HC<SCD | - | - | - | 3.91 | 0.048 | 0.970 | 4.02 | 0.009 | 0.962 |
|  | HC>SCD | - | - | - | 4.56 | 0.005 | 0.687 | 5.00 | **<0.001** | 0.207 |
|  | HC<MCI | - | - | - | 4.28 | 0.047 | 0.993 | 4.49 | 0.007 | 0.799 |
|  | HC>MCI | - | - | - | 4.92 | 0.024 | 0.867 | 4.90 | 0.006 | 0.472 |
|  | SCD>MCI | - | - | - | 4.18 | 0.008 | 0.682 | 4.66 | **<0.001** | 0.446 |
|  | SCD<MCI | - | - | - | 5.85 | 0.003 | 0.380 | 5.38 | 0.006 | 0.437 |
| SVF semantic cluster size | HC<SCD | - | - | - | 3.67 | 0.046 | 0.857 | 4.62 | **<0.001** | 0.300 |
|  | HC>SCD | - | - | - | 4.46 | 0.004 | 0.722 | 4.89 | **<0.001** | 0.360 |
|  | HC<MCI | - | - | - | 4.44 | 0.026 | 0.966 | 4.08 | 0.022 | 0.786 |
|  | HC>MCI | - | - | - | 4.31 | 0.028 | 0.981 | 3.81 | 0.020 | 0.706 |
|  | SCD>MCI | - | - | - | 3.83 | 0.021 | 0.762 | 4.83 | 0.008 | 0.568 |
|  | SCD<MCI | - | - | - | 4.95 | 0.008 | 0.648 | 4.21 | 0.003 | 0.440 |
| SVF semantic cluster switches | HC<SCD | - | - | - | 4.45 | 0.024 | 0.630 | 6.04 | **<0.001** | **0.023** |
|  | HC>SCD | - | - | - | 3.79 | 0.034 | 0.946 | 4.20 | 0.020 | 0.823 |
|  | HC<MCI | - | - | - | 4.38 | 0.008 | 0.778 | 4.50 | **<0.001** | 0.440 |
|  | HC>MCI | - | - | - | 3.85 | 0.028 | 0.932 | 4.41 | **<0.001** | 0.822 |
|  | SCD>MCI | - | - | - | 5.07 | 0..006 | 0.307 | 5.07 | **<0.001** | 0.780 |
|  | SCD<MCI | - | - | - | 4.60 | 0.003 | 0.568 | 4.61 | 0.007 | 0.440 |
| SVF temporal cluster size | HC<SCD | - | - | - | 4.98 | 0.006 | 0.864 | 4.00 | 0.025 | 0.957 |
|  | HC>SCD | - | - | - | 4.02 | 0.028 | 0.943 | 4.16 | 0.038 | 0.987 |
|  | HC<MCI | - | - | - | 4.72 | 0.042 | 0.950 | 5.37 | 0.045 | 0.966 |
|  | HC>MCI | - | - | - | 4.74 | 0.021 | 0.860 | 4.31 | 0.063 | 0.993 |
|  | SCD>MCI | - | - | - | 4.95 | 0.009 | 0.750 | 5.36 | 0.026 | 0.853 |
|  | SCD<MCI | - | - | - | 4.80 | 0.098 | 0.868 | 4.74 | 0.090 | 0.866 |
| SVF temporal cluster switches | HC<SCD | - | - | - | 4.25 | 0.010 | 0.699 | 3.93 | 0.023 | 0.906 |
|  | HC>SCD | - | - | - | 4.44 | 0.009 | 0.726 | 4.76 | 0.008 | 0.790 |
|  | HC<MCI | - | - | - | 4.34 | 0.003 | 0.593 | 4.64 | 0.010 | 0.275 |
|  | HC>MCI | - | - | - | 5.07 | **<0.001** | 0.271 | 4.45 | 0.005 | 0.754 |
|  | SCD>MCI | - | - | - | 5.09 | **<0.001** | 0.694 | 6.10 | **<0.001** | 0.072 |
|  | SCD<MCI | - | - | - | 4.08 | 0.004 | 0.706 | 5.30 | **<0.001** | 0.952 |
| VLT delayed recall, correct count | HC<SCD | 3.96 | 0.020 | 0.987 | - | - | - | - | - | - |
|  | HC>SCD | 4.36 | 0.034 | 0.995 | - | - | - | - | - | - |
|  | HC<MCI | 4.67 | 0.060 | 0.990 | - | - | - | - | - | - |
|  | HC>MCI | 4.44 | 0.009 | 0.786 | - | - | - | - | - | - |
|  | SCD>MCI | 3.67 | **<0.001** | 0.458 | - | - | - | - | - | - |
|  | SCD<MCI | 3.80 | 0.006 | 0.664 | - | - | - | - | - | - |
| VLT delayed recall serial cluster size | HC<SCD | 4.38 | 0.003 | 0.404 | - | - | - | - | - | - |
|  | HC>SCD | 3.72 | 0.092 | 0.989 | - | - | - | - | - | - |
|  | HC<MCI | 4.47 | 0.060 | 0.968 | - | - | - | - | - | - |
|  | HC>MCI | 4.17 | 0.022 | 0.897 | - | - | - | - | - | - |
|  | SCD>MCI | 4.85 | **<0.001** | 0.368 | - | - | - | - | - | - |
|  | SCD<MCI | 5.84 | **<0.001** | 0.542 | - | - | - | - | - | - |
| VLT delayed recall midlist items | HC<SCD | 3.54 | 0.040 | 0.999 | - | - | - | - | - | - |
|  | HC>SCD | 3.74 | 0.023 | 0.981 | - | - | - | - | - | - |
|  | HC<MCI | 4.02 | 0.009 | 0.941 | - | - | - | - | - | - |
|  | HC>MCI | 3.90 | **<0.001** | 0.628 | - | - | - | - | - | - |
|  | SCD>MCI | 4.90 | **<0.001** | 0.796 | - | - | - | - | - | - |
|  | SCD<MCI | 4.84 | **<0.001** | 0.442 | - | - | - | - | - | - |
| VLT delayed recall primacy items | HC<SCD | 4.12 | 0.006 | 0.736 | - | - | - | - | - | - |
|  | HC>SCD | 4.49 | 0.006 | 0.749 | - | - | - | - | - | - |
|  | HC<MCI | 3.60 | **<0.001** | 0.968 | - | - | - | - | - | - |
|  | HC>MCI | 3.82 | 0.020 | 0.954 | - | - | - | - | - | - |
|  | SCD>MCI | 4.31 | 0.026 | 0.629 | - | - | - | - | - | - |
|  | SCD<MCI | 4.45 | 0.003 | 0.385 | - | - | - | - | - | - |
| VLT delayed recall recency items | HC<SCD | 3.69 | 0.034 | 0.983 | - | - | - | - | - | - |
|  | HC>SCD | 4.91 | 0.004 | 0.975 | - | - | - | - | - | - |
|  | HC<MCI | 3.55 | 0.025 | 0.986 | - | - | - | - | - | - |
|  | HC>MCI | 4.08 | 0.030 | 0.986 | - | - | - | - | - | - |
|  | SCD>MCI | 4.28 | **<0.001** | 0.667 | - | - | - | - | - | - |
|  | SCD<MCI | 4.92 | **<0.001** | 0.552 | - | - | - | - | - | - |

## **Table S7** Brain regions showing significant associations between resting-state network connectivity and digital speech features based on the seed-based approach

| **Digital speech features** | **Seed** | **Cluster size (voxels)** | **mean T** | **peak MNI Coordinates** | | | **peak T** | **Side** | **Anatomical location** |
| --- | --- | --- | --- | --- | --- | --- | --- | --- | --- |
|  |  |  |  |  |  |  |  |  |  |
| SB-C global score | IFG | 20 | 2.66 | 6 | 51 | 36 | 3.14 | R | Superior medial frontal gyrus |
|  | dlPFC | 107 | 3.15 | -63 | -33 | 27 | 4.90 | L | Supramarginal gyrus |
|  |  | 32 | 2.60 | 63 | -39 | 39 | 2.94 | R | Supramarginal gyrus |
|  |  | 52 | 2.88 | -39 | 45 | 27 | 3.80 | L | Middle frontal gyrus |
|  | anPFC | 20 | 2.64 | 39 | 36 | 39 | 3.32 | R | Middle frontal gyrus |
|  |  | 45 | 2.93 | -36 | 33 | 30 | 3.76 | L | Middle frontal gyrus |
|  |  | 20 | 2.52 | 54 | -39 | 48 | 2.79 | R | Inferior parietal lobule |
| SB-C executive score | IFG | 20 | 2.96 | 51 | 6 | -27 | 3.69 | R | Middle temporal gyrus |
|  | rPCC | 25 | 2.79 | 60 | -3 | -24 | 3.33 | R | Middle temporal gyrus |
|  | dlPFC | 17 | 2.76 | 36 | 18 | 3 | 3.16 | R | Insula |
|  |  | 47 | 2.81 | 48 | -27 | 42 | 3.56 | R | Postcentral gyrus |
|  |  | 22 | 2.65 | -21 | 3 | 54 | 3.33 | L | Superior frontal gyrus |
|  | anPFC | 22 | 2.98 | 63 | -45 | 18 | 3.69 | R | Superior temporal gyrus |
|  |  | 21 | 2.82 | 27 | 42 | 33 | 3.96 | R | Superior frontal gyrus |
| SB-C memory score | mPFC | 22 | 2.57 | -24 | -84 | -36 | 2.88 | L | Cerebellum Crus II |
|  | dlPFC | 74 | 2.37 | -42 | 42 | 24 | 3.90 | L | Middle frontal gyrus |
|  |  | 24 | 2.15 | 39 | 51 | 21 | 3.84 | R | Middle frontal gyrus |
|  |  | 120 | 2.16 | 60 | -42 | 36 | 3.18 | R | Supramarginal gyrus |
|  |  | 125 | 2.73 | -63 | -33 | 27 | 4.30 | L | Supramarginal gyrus |
|  |  | 22 | 2.23 | 6 | -24 | 42 | 3.06 | R | Middle cingulate cortex |
|  | anPFC | 20 | 2.77 | -36 | 33 | 30 | 3.56 | L | Middle frontal gyrus |
|  |  | 40 | 2.72 | 51 | -48 | 51 | 3.47 | R | Inferior parietal lobule |
| SB-C processing speed score | mPFC | 21 | 2.58 | -18 | -90 | -36 | 3.10 | L | Cerebellum Crus II |
|  | dlPFC | 85 | 3.08 | -51 | -42 | 33 | 4.41 | L | Supramarginal gyrus |
|  |  | 40 | 2.85 | -45 | 42 | 18 | 3.69 | L | Middle frontal gyrus |
|  | anPFC | 22 | 2.85 | -36 | 30 | 30 | 3.47 | L | Middle frontal gyrus |
| SVF correct count | IFG | 23 | 2.70 | -6 | 54 | 30 | 3.29 | L | Superior medial frontal gyrus |
|  |  | 34 | 2.84 | -21 | 51 | 30 | 3.52 | L | Superior frontal gyrus |
|  |  | 20 | 2.81 | 6 | 51 | 36 | 3.58 | R | Superior medial frontal gyrus |
|  | dlPFC | 20 | 2.75 | 42 | 48 | 21 | 3.68 | R | Middle frontal gyrus |
|  | anPFC | 44 | 2.74 | 27 | 42 | 33 | 4.34 | R | Superior frontal gyrus |
|  |  | 24 | 2.94 | 66 | -42 | 18 | 4.33 | R | Superior temporal gyrus |
|  |  | 42 | 2.85 | -45 | 33 | 30 | 3.84 | L | Middle frontal gyrus |
| SVF word frequency | IFG | 32 | 2.77 | -6 | 57 | 18 | 3.64 | L | Superior medial frontal gyrus |
|  |  | 33 | 3.00 | 6 | 51 | 39 | 4.80 | R | Superior medial frontal gyrus |
|  | anPFC | 85 | 2.79 | 51 | -27 | 27 | 3.98 | R | Inferior parietal lobule |
| SVF semantic cluster size | dlPFC | 42 | 2.67 | -24 | 0 | 51 | 3.07 | L | Superior frontal gyrus |
|  | anPFC | 32 | 2.82 | -30 | 33 | 33 | 3.30 | L | Superior frontal gyrus |
|  |  | 28 | 2.93 | 27 | 27 | 42 | 4.12 | R | Middle frontal gyrus |
| SVF semantic cluster switches | IFG | 28 | 2.59 | -39 | 9 | 42 | 3.00 | L | Precentral gyrus |
|  | dlPFC | 42 | 2.65 | 51 | -54 | 51 | 3.38 | R | Inferior parietal lobule |
|  |  | 24 | 2.98 | -27 | -54 | -33 | 3.94 | L | Cerebellum Lobule VI |
| SVF temporal cluster size | IFG | 20 | 2.72 | 48 | 15 | -30 | 3.23 | R | Middle temporal pole |
|  | dlPFC | 53 | 2.89 | 63 | -24 | 42 | 3.08 | R | Supramarginal gyrus |
|  |  | 21 | 2.74 | -48 | -33 | 48 | 3.55 | L | Postcentral gyrus |
|  |  | 27 | 2.64 | 30 | -3 | 54 | 3.12 | R | Middle frontal gyrus |
|  |  | 48 | 3.05 | -24 | 0 | 54 | 4.50 | L | Superior frontal gyrus |
|  |  | 33 | 3.10 | 18 | -69 | 57 | 4.49 | R | Superior parietal lobule |
| SVF temporal cluster switches | IFG | 37 | 2.95 | -3 | 21 | 54 | 3.65 | L | Supplementary motor area |
|  | anPFC | 67 | 3.16 | -27 | 54 | 21 | 5.23 | L | Superior frontal gyrus |
|  |  | 40 | 2.88 | 27 | 57 | 24 | 3.90 | R | Superior frontal gyrus |
| VLT delayed recall, correct count | mPFC | 38 | 2.84 | -24 | -81 | -39 | 3.72 | L | Cerebellum Crus II |
|  |  | 20 | 2.82 | -3 | 51 | 30 | 3.43 | L | Superior medial frontal gyrus |
|  |  | 40 | 2.77 | 6 | 48 | 30 | 3.46 | R | Superior medial frontal gyrus |
| VLT delayed recall primacy items | mPFC | 25 | 2.84 | 9 | 57 | 36 | 4.50 | R | Superior medial frontal gyrus |
| VLT delayed recall midlist items | rPCC | 21 | 2.81 | 3 | -27 | 42 | 3.35 | R | Middle cingulate cortex |
|  | mPFC | 28 | 2.90 | -33 | -78 | -33 | 3.95 | L | Cerebellum Crus I |
| VLT delayed recall serial cluster size | dlPFC | 37 | 3.04 | 27 | 54 | 27 | 4.57 | R | Middle frontal gyrus |

Uncorrected threshold of p < .01, with cluster size ≥ 20 voxels.

## **Fig. S2**Group-level RSNs maps generated via the seed-based approach.


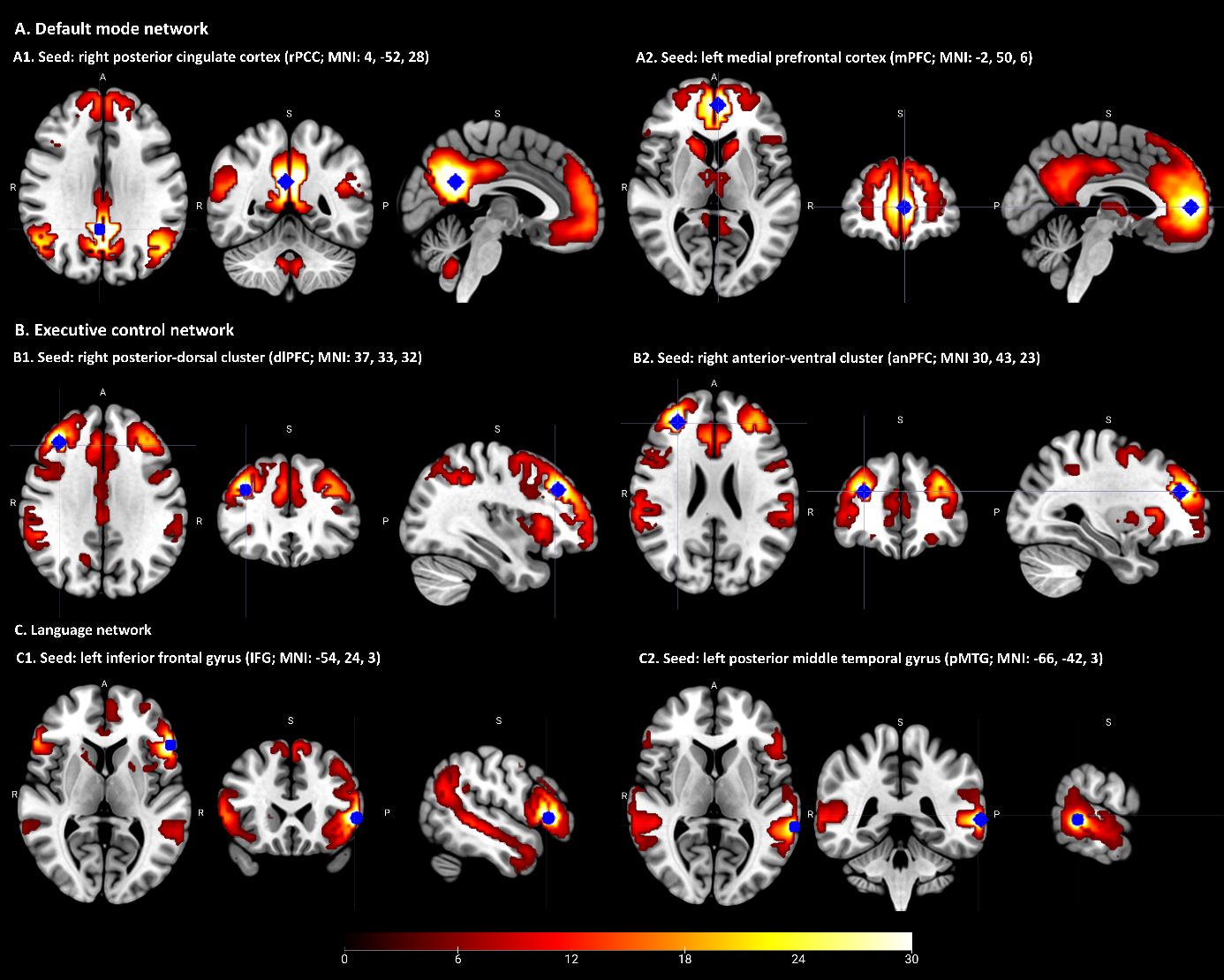


Spatial maps of the (A) default mode network (DMN), (B) executive control network (ECN), and (C) language network (LAN) identified using a seed-based approach with a 4 mm smoothing kernel. Each network is visualized by displaying clusters that survive the FWE correction threshold of p < .05, with a minimum cluster size of 20 voxels, derived from group-level RSN maps. Blue voxels mark the seed regions, while warm-colored clusters (color scale representing t-values) represent the group-level RSNs. These thresholded maps were binarized to create masks for subsequent regression analyses. All results are shown in MNI space over a standard anatomical template. Images are shown in radiological convention (left = right hemisphere).
